# Influenza Illness in Pregnant Indian Women: A Cross-Sectional Study

**DOI:** 10.1155/2016/1248470

**Published:** 2016-01-19

**Authors:** Parvaiz A. Koul, Nargis K. Bali, Hyder Mir, Farhat Jabeen, Abida Ahmad

**Affiliations:** ^1^Department of Internal & Pulmonary Medicine, Sher-I-Kashmir Institute of Medical Sciences, Soura, Srinagar 190011, India; ^2^Department of Clinical Microbiology, Sher-I-Kashmir Institute of Medical Sciences, Soura, Srinagar 190011, India; ^3^Department of Obstetrics and Gynecology, Lalla Ded Hospital for Women, Government Medical College, Srinagar 190010, India; ^4^Department of Obstetrics and Gynecology, Sher-I-Kashmir Institute of Medical Sciences, Soura, Srinagar 190011, India

## Abstract

Data about burden of influenza in pregnancy in India are scant. In order to assess the contribution of influenza to acute respiratory illness (ARI) in pregnancy, 266 north Indian pregnant females with febrile ARI were studied from December 2014 to May 2015. Twin nasopharyngeal/oropharyngeal swabs were obtained and tested for influenza viruses by RT-PCR. Fifty (18.8%) patients tested positive for influenza (A/H1N1pdm09 in 41, A/H3N2 in 8, and influenza B Yamagata in 1). Rigors, headache, and a family history of ARI were significantly more frequent in influenza positive patients. Oseltamivir and supportive therapy were administered to all confirmed cases. Nine influenza positive cases needed hospitalization for their respiratory illness, and 5 developed respiratory failure. Of these, 4 (3 in third trimester) succumbed to their illness. We conclude that influenza viruses are a cause of significant morbidity and mortality among pregnant females with ARI in north India. As such, appropriate preventive strategies of influenza vaccination and early initiation of antiviral therapy during illness are stressed.

## 1. Introduction

Influenza during pregnancy has been associated with considerable morbidity and mortality. Pregnant women were observed to be at high risk of complications such as pneumonia and death during the influenza pandemics of 1918, 1957, and 2009. In the 1918 pandemic, Harris described an overall mortality of 27% among pregnant females who developed influenza-associated pneumonia which exceeded 50% in the third trimester of pregnancy [1]. In the 1957 pandemic, there was increased mortality due to influenza complicating pregnancy as compared to nonpregnant females [2]. Pregnant women accounted for about 6% of influenza related hospitalizations, ICU admissions, and deaths in the 2009 influenza pandemic, even though they constitute only 1% of the US population at any point in time [3, 4]. In the 18–29 years age group, pregnancy accounted for up to 29% of influenza-associated hospitalizations and 16% of deaths [5–7]. About 50% of the pregnancy-associated deaths in the April–September 2009 H1N1 pandemic period were observed in the third trimester whereas 36% occurred during the second trimester [8]. In a recent review of 100 studies published between 1961 and 2015, investigators reported that, compared to the general population, pregnant women are more often hospitalized and admitted to an intensive care unit due to influenza virus infection [9]. During May-June 2009, pregnant women were 7.2 times more likely to be hospitalized and 4.3 times more likely to be admitted to an ICU than nonpregnant women [10]. Coexiting conditions such as asthma or diabetes put pregnant women at 3-4 times greater risk of morbidity as compared to nonpregnant control subjects with similar high-risk conditions [11].

Influenza has a significant impact on the mother as well as the fetus. Infection during pregnancy has been associated with an approximately fivefold increase in perinatal mortality, including miscarriages, stillbirths, and early neonatal diseases and death [11, 12]. A 3-fold increased risk of premature and complicated birth was observed in pregnant women hospitalized with A/H1N1pdm09 [13].

There are limited data describing the burden of influenza in pregnancy in India. Previously, Gunasekaran et al. found that a higher proportion of pregnant women were positive for both seasonal influenza (11.1% pregnant women versus 1.4% nonpregnant women) and influenza A/H1N1pdm09 (21.4% pregnant women versus 2.7% nonpregnant women) [14]. However, Pramanick et al. reported that, among all women presenting with ILI/SARI, influenza A (pH1N1) was positive in 25.3% of pregnant/puerperal women and 29.6% of nonpregnant women [15]. In spite of high rate of morbidity and mortality, uptake of influenza vaccination in pregnant females is very low [16]. Therefore, it is imperative to improve our understanding of the burden of influenza viruses to respiratory illness in pregnancy so that preventive measures such as vaccination can be rationally implemented.

## 2. Material and Methods

The study was conducted in 2 tertiary care referral centers for obstetrics cases of the Kashmir Valley in the north Indian state of Jammu & Kashmir in a cross-sectional design. We have previously documented a temperate seasonality of influenza circulation with wintertime peaks in this region with a significant contribution of influenza towards causation of respiratory illness [17]. About 350 to 400 women seeking obstetric care are seen in the Lalla Ded (LD) Hospital for Women whereas about 150 women were seen in Sher-I-Kashmir Institute of Medical Sciences (SKIMS). These hospitals cater to the majority of the institutional obstetrics cases in the valley. The sample population comprised women (≥18 years) presenting in any stage of pregnancy to the obstetrics departments with symptoms of influenza-like illness (ILI) (defined as a sudden onset of fever ≥38°C, with cough or sore throat in the absence of any other diagnosis and an onset within the past 10 days) or severe acute respiratory infection (SARI) (defined as a patient with ILI who required admission for the respiratory illness). Patients without fever were labeled as acute respiratory illness (ARI).

### 2.1. Sample Collection and Testing

The study started in December 2014 and continued until May 2015 when the enrollment target was achieved. Clinical history and examination of the study patients were recorded for all participants including any history of clustering (two or more cases that were related in time and space, e.g., in a home or workplace) for the entire study period. Parity and obstetric history were recorded along with any history of complications of the pregnancy. Combined throat and nasal swabs were collected in viral transport medium at the LD Hospital/SKIMS and transported immediately to the Influenza Laboratory at SKIMS. All samples were processed within 3-4 hours of collection. Samples were tested by real-time RT-PCR for influenza viruses A and B using the standard CDC protocol [18]. All influenza A positive samples were further subtyped using primers and probes for A/H1N1pdm09 and A/H3. Influenza B viruses were further subtyped into B/Yamagata and B/Victoria subtypes using specific primers.

A confirmed case of influenza was defined as a study participant with ILI/SARI/ARI with laboratory-confirmed influenza A or B detected by RT-PCR.

### 2.2. Analysis

Statistical analysis of the data was performed using SPSS statistical software version 11.0, IBM Corp., USA. The clinical features of influenza positive and influenza negative patients were compared. Data have been expressed as Mean ± SD. Categorical variables were compared using Fisher's exact/Chi-square test and continuous variables by employing Student's *t*-test. A *p* value of <0.05 was considered significant.

### 2.3. Ethics

The study was approved by the Institute Ethics Committee of Sher-I-Kashmir Institute of Medical Sciences, Srinagar (protocol ID: RP 241/2014 of 2014).

## 3. Results

The 266 observed women were aged 18–39 years (median 27 years), 257 of which had ILI whereas 9 required hospitalization for their respiratory illness. While 141 patients were from the urban setting, 125 were from rural areas. The patients presented within 1–10 (median 4) days of the onset of symptoms that included fever, chills/rigors, cough, nasal discharge, sore throat, headache, and body aches. The parity status of the women was primigravida (*n* = 103), G2 (*n* = 71), G3 (*n* = 57), G4 (*n* = 22), G5 (*n* = 10), G6 (*n* = 2), and G7 (*n* = 1). The duration of pregnancy ranged from 5 weeks to 9 months (median 27.5 weeks); 144 (66.7%) belonged to the third trimester, 94 (43.5%) to the second trimester, and 28 (12.9%) to the first trimester. Eleven patients had a history of recurrent pregnancy loss and 55 had a history of previous cesarean section. Comorbid illnesses included pregestational/gestational diabetes (*n* = 11), gestational hypertension (*n* = 23), anaemia (*n* = 120), and hypothyroidism (*n* = 3). One of the patients had a twin pregnancy and one had a large-for-gestation baby. None had received influenza vaccination in the current pregnancy and or had been advised to receive it.

Fifty (18.8%; age 20–35 years; median, 20) of the 266 observed patients tested positive for influenza viruses against the routine positivity of around 18% seen over a 5-year period of surveillance [17]. Further subtyping of the isolates revealed that 41 (82%) were positive for the A/H1N1pdm09, 8 (16%) were positive for A (H3N2), and 1 (2.0%) was positive for influenza B (Yamagata lineage). Of the 50 patients who tested positive for influenza viruses, 30 (60%) were in the third trimester, 15 (30%) were in the second, and 5 (10%) were in the first. The relative positivity did not differ among the 3 trimesters, ranging from 15.9% to 20.8% (*p* > 0.05). Various symptoms reported by influenza positive patients included fever with rigors (*n* = 48), cough (*n* = 47), body aches (*n* = 47), fatigue (*n* = 47), headache (*n* = 45), nasal discharge (*n* = 40), breathlessness (*n* = 40), sore throat (*n* = 41), expectoration (*n* = 30), vomiting (*n* = 8), and diarrhea (*n* = 5). Sixty-two of the observed patients (44% of all influenza positive patients) had a history of an ARI/ILI in the family. Comorbidities in influenza positive patients included pregestational/gestational hypertension (*n* = 6), pregestational/gestational diabetes (*n* = 3), and anemia (*n* = 26). The frequency of comorbidities like hypertension, diabetes, or anemia was not significantly different in influenza positive as compared to influenza negative participants (*p* = 0.35, 0.46, and 0.41, resp.). A comparative analysis of clinical presentations among influenza positive and influenza negative patients is given in Table 1. Rigors, headache, and nasal discharge were seen significantly more frequently in patients who tested influenza positive.

**Figure 1:**
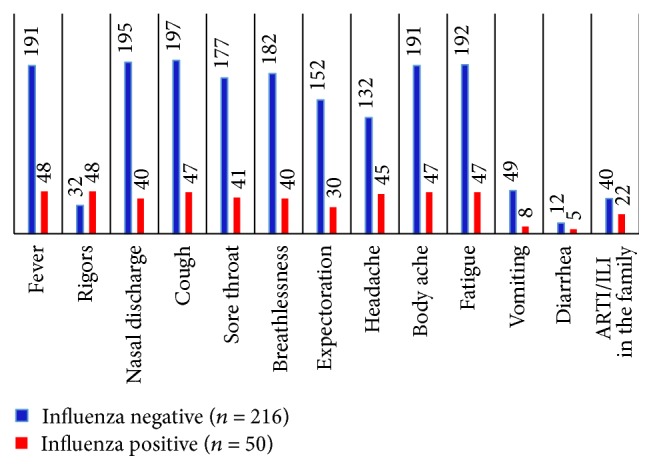
Graphical representation of the major symptoms at presentation.

All patients who were influenza positive were administered oral Oseltamivir. Of the 50, nine required admission for severe respiratory illness (bilateral infiltrates with respiratory failure (*n* = 5) and lobar pneumonia (*n* = 4) (Figure 1)). Five of these required invasive mechanical ventilation for respiratory failure, broad spectrum antibiotics, and vasopressor agents. Four patients succumbed to multiorgan failure. Three of these were in the third trimester whereas one was in the second trimester. These patients had presented with respiratory symptoms of 2–5 days, were aged 21–29 years, and developed severe respiratory distress prior to presentation to the hospital. All were previously healthy and had no attendant comorbidities, and A/H1N1pdm09 was detected in all the 4. A premortem cesarean section in one patient led to the survival of the baby. Apart from these 4, all patients had an uncomplicated recovery.

## 4. Discussion

Our data suggest that influenza viruses are an important cause of respiratory illness in pregnant females with considerable morbidity and mortality. To the best of our knowledge, this study is the first one to document the burden of influenza in pregnant females in India employing active surveillance in pregnant women.

Influenza virus contributed to 18.8% of the acute respiratory infections in the observed pregnant females with 82% of the infections caused by the influenza A/H1N1pdm09, 16% by A/H3N2, and 2% by influenza B virus. The comparative prevalence of the various strains in the community surveillance included A/H1N1pdm09 (86%), A/H3N2 (12%), and B (Yamagata) 1% (unpublished data), the strain distribution among the pregnant females being similar to the general community trends of circulation. While only two previous Indian reports exist of seasonal influenza viruses causing respiratory illness in pregnancy [14, 15], others have focused on pandemic A/H1N1pdm09 virus [19–23]. Predominance of A/H1N1pdm09 as the dominant virus causing respiratory illness in pregnant females was also reported earlier [14, 15]. However, we attribute this predominance of the strain to the general circulation of A/H1N1pdm09 during the season rather than any specific propensity of pregnant females to A/H1N1pdm09 as the strain was the major circulating strain at the time of the study (Koul PA. unpublished data).

The risk of hospitalization in pregnant women has been observed to be 18-fold compared to nonpregnant women even during the interpandemic period, with risk being greatest among women in later stages of pregnancy [24–26]. The majority (60%) of influenza cases in our study also occurred in the third trimester. Four of the 5 pregnant women who developed respiratory failure were in the third trimester of their pregnancy and 3 of them died. The higher risk of severe disease and ICU admission is consistent with earlier reports [2, 20–22, 27], with high rates of complications such as pneumonia and renal failure. Pregnant women once infected seem to develop severe infection [28]. The case fatality rate in our study was 8%, all being A/H1N1pdm09 positive. A/H1N1pdm09 influenza infection has been associated with higher mortality (25–70%) among Indian pregnant women [21, 23, 29] than that reported from other countries [30, 31]. Such high figures could be an overestimation as the total number of pregnant females in these studies is low. Different sampling methods and diverse study population could be other factors attributing to such high figures and there could be a contribution of relatively inadequate ICU facilities. However, Gunasekaran et al. reported a mortality of only 3.7% [14]. While associated comorbidities like asthma and diabetes pose a higher risk of morbidity in pregnant females as compared to their nonpregnant controls [11, 24], none of our patients with adverse outcomes had a comorbid illness.

The mechanisms that increase the risk of serious complications from influenza in pregnancy are incompletely understood. A combination of cardiac, respiratory, hormonal, and immunological changes accompany pregnancy which impair responses to infection and increase the likelihood of serious complications that require admission to the hospital [32, 33]. These changes include elevation of the diaphragm due to increased uterine size, increased intra-abdominal pressure, increased respiratory rate, reduced chest compliance, and high risk of aspiration as a consequence. Other physiological changes include reduced tidal volume and lung capacity and increased cardiac output and oxygen consumption. Decreases in adaptive immunity seen in later stages of pregnancy is consistent with the observed increase in the severity of certain infectious diseases during later pregnancy. Decreases in the numbers and function of CD4+, CD8+, and natural killer cells could affect antimicrobial responses and delay clearance of the infecting microorganism [34]. Additionally, while some cytokines are suppressed (e.g., IFN*γ* and VEGF), others (e.g., the proinflammatory cytokines TNF*α* and G-CSF) are increased throughout pregnancy. The cytokine changes result in interplay with resultant changes in Th1 and Th2 responses, NK cell function, and antigen presentation. When incubated with influenza viruses, peripheral blood mononuclear cells from third trimester pregnant women exhibit reduced antiviral gene expression and consequently higher replication of the viruses [35].

An important observation of the current study was that none of the pregnant females had received influenza vaccination despite being pregnant in the influenza season. Although routine vaccination of pregnant females against influenza with inactivated trivalent influenza vaccine is recommended [36], it has been only recently endorsed by the Federation of Obstetricians and Gynecologists of India (FOGSI) [37]. We have recently demonstrated a poor uptake of influenza vaccination among 1000 pregnant females in Kashmir where vaccinations were not adopted at all [16]. In absence of similar data from other parts of the country, these data more or less reflect the vaccination patterns across the country. Importantly, major deficits in the knowledge, attitude, and practices regarding vaccination against influenza have been observed in the healthcare providers [16, 38] who are supposed to be the prescribers of vaccination to pregnant females. Maternal immunization during the period of influenza virus circulation has been associated with statistically significant reductions in febrile respiratory illness among mothers and infants, higher mean birth weight in infants, and lower proportion of infants who were of small-for-gestation age [39], without any attributable adverse fetal, perinatal, or infant outcomes [40]. Immunologic responses generated by influenza immunization are comparable in pregnant and nonpregnant women and can provide protection to the fetus and infant by transferring specific antibodies across the placenta [41] and in breast milk [42]. However, despite ample evidence to the contrary, misperceptions regarding the safety and efficacy of influenza vaccination are common among health care providers in India and advocate effective educational interventions.

Our study likely represents an underestimate of the total number of pregnant females with influenza during the period under study and an overestimate of the proportion of pregnant women with severe illness. Influenza positive women in the early stages might not have been aware of the status of their pregnancy and might not as such have reported the same. In addition, the lack of the data regarding outcomes of pregnancy is a limitation of the current study. However, these data provide the first evidence of the burden of influenza illness in pregnancy in India and provide strong motivation for additional research, particularly on birth outcomes. The data also emphasize the need for improved awareness among healthcare professionals regarding influenza vaccination during pregnancy and early initiation of antiviral therapy when influenza infection is suspected.

## Figures and Tables

**Table 1:** Clinical features of influenza positive and influenza negative patients.

Clinical features	Influenza negative *N* = 216 *N* (%)	Influenza positive *N* = 50 *N* (%)	*p* value
Duration of symptoms (mean ± SD range) in days; median	4.25 ± 1.98 (1–10); 4	3.74 ± 2.03 (1–10); 4	0.10
Fever	191 (88.4)	48 (96)	0.11
Rigors	32 (14.8)	48 (96)	<0.0001
Nasal discharge	195 (90.3)	40 (80)	0.041
Ear discharge	1 (0.5)	0 (0)	
Cough	197 (91.2)	47 (94)	0.52
Sore throat	177 (81.9)	41 (82)	0.98
Breathlessness	182 (84.3)	40 (80)	0.46
Expectoration	152 (70.4)	30 (60)	0.15
Headache	132 (61.1)	45 (90)	0.0001
Body ache	191 (88.4)	47 (94)	0.25
Fatigue	192 (88.9)	47 (94)	0.28
Vomiting	49 (22.7)	8 (16)	0.30
Diarrhea	12 (5.6)	5 (10)	0.25
Seizures	1 (0.5)	0 (0)	
ARTI/ILI in the family	40 (15.0)	22 (44)	<0.0001

ARI = acute respiratory illness; ILI = influenza-like illness.
